# Peripheral joint ankylosis in the spontaneous model of arthritis in DBA/1 mice is genetically associated with BMP signaling

**DOI:** 10.1186/1546-0096-9-S1-P314

**Published:** 2011-09-14

**Authors:** S Carter, I Derese, K Braem, AM Valdes, FP Luyten, RJ Lories

**Affiliations:** 1 Laboratory for Skeletal Development and Joint Disorders, Division of Rheumatology, KU Leuven, Belgium; 2 Department of Twin Research and Genetic Epidemiology, Kings College London, London, UK

## 

Progression of ankylosis in patients with ankylosing spondylitis is highly variable. This suggests that ankylosis is at least partially due to genetic factors. We used the ankylosing enthesits model in DBA/1 mice to search for associated genes.

Male DBA/1 were crossed with female BALB/c mice. Male F2 mice from different litters were studied by histomorphology at 26 weeks. 159 markers with sequence-length polymorphisms on the autosomes were selected. Median spacing between the markers was 6.9 cM. 162 F2 male mice were studied. Genes in regions of interests were linked to skeletal development, bone morphogenetic protein (BMP) and Wnt signaling pathways with the Gene Ontology database. The association was evaluated by Chi-square tests with a False Discovery Rate (FDR) algorithm.

Incidence of ankylosing enthesitis was lower in the F2 generation as compared to wild-type DBA/1 males (42% vs. 72%; p<0.0001). When applying the FDR algorithm for 159 markers, associations with *D3MIT199* and *D3MIT160* were significant (p<0.016). Adjacent markers were additionally genotyped. In the associated region between markers *D3MIT42* and *D3MIT129*, 162 genes were found among which *Bmpr1b*, *Cxxc4*, *Lef1*, *Papss1*, *Pitx2*, and *Ube2d3*. Only BMP receptor type 1b (*BMPr1b*) was specifically upregulated in mice with spontaneous arthritis as demonstrated by quantitative RT-PCR.

By using F2 mouse genetics in the analysis of ankylosis, we identified a locus on chromosome 3 that shows association. Within this locus, several genes could play a role in ankylosis but the expression profile suggests that BMP receptor 1b is involved, further supporting a critical role for BMPs in this process.

